# Injectable Platelet-Rich Fibrin and Advanced Platelet-Rich Fibrin Demonstrate Enhanced Anti-Biofilm Effect Compared to Enamel Matrix Derivatives on Decontaminated Titanium Surfaces

**DOI:** 10.3390/dj12060175

**Published:** 2024-06-06

**Authors:** Jothi Varghese, Liza L. Ramenzoni, Padmaja A. Shenoy, Patrick R. Schmidlin, Shubhankar Mehrotra, Vinayak Kamath

**Affiliations:** 1 Department of Periodontology, Manipal College of Dental Sciences, Manipal Academy of Higher Education, Manipal 576104, Karnataka, India; jothimv@gmail.com (J.V.); shubhankarmehrotra26@gmail.com (S.M.); 2 Clinic of Conservative and Preventive Dentistry, Center of Dental Medicine, University of Zurich, Plattenstrasse 11, 8032 Zurich, Switzerland; 3 Department of Microbiology, Kasturba Medical College, Manipal Academy of Higher Education, Manipal 576104, Karnataka, India; padmaja.shenoy@manipal.edu; 4 Department of Public Health Dentistry, Goa Dental College and Hospital, Bambolim 403201, Goa, India; b_vk2002@yahoo.com

**Keywords:** i-PRF, A-PRF+, EMD, titanium implant, biofilm, antimicrobial

## Abstract

Background: The search for effective antimicrobial agents to mitigate peri-implant infections remains a crucial aspect of implant dentistry. This study aimed to evaluate and compare the antimicrobial efficacy of i-PRF, A-PRF+, and enamel matrix derivative (EMD) on decontaminated rough and smooth titanium (Ti) discs. Materials and Methods: Rough and smooth Ti discs were coated with multispecies biofilm and thoroughly debrided using a chitosan-bristled brush. Subsequently, i-PRF, A-PRF+, and EMD were applied. Untreated discs served as control. Residual adherent bacteria present on the treated Ti discs were visualized by SEM and quantified using culture technique, and colony-forming units (CFUs) were measured after 48 h and 7 days. Results: i-PRF demonstrated better antimicrobial effectiveness on both smooth and rough implant surfaces as compared to A-PRF+ and EMD (*p* < 0.001). In all the experimental groups, smooth Ti discs displayed a greater reduction in microbes compared to rough Ti discs when treated with the biologics. The major reduction in CFU values was determined after seven days. Conclusions: i-PRF as a regenerative material may also be suitable for decontaminating implant surfaces, which could influence tissue healing and regenerative outcomes positively.

## 1. Introduction

Dental implants represent a viable therapeutic modality for restoration of missing teeth with high and predictable success rates. However, they are susceptible to bacterially induced peri-implant inflammation and, as a consequence, to peri-implant pocket formation and bone loss [1,2]. According to a recent systematic review, the prevalence of peri-implantitis remains variable, with rates ranging from 12.53% to 19.53% depending on clinical cases and selected outcome parameters [3]. A combination of non-surgical and surgical methods has been employed to create an effective and reliable treatment strategy for peri-implantitis, with bacterial decontamination being a key component accompanied with regeneration of the lost peri-implant tissue [4]. Most of the on-going research is directed towards effective treatment protocols for decontaminating implant surfaces. Investigators have employed a variety of mechanical and chemical agents as well as combinations thereof to eliminate microbes from the implant surfaces. Mechanical therapy using curettes has demonstrated limited ability in elimination of bacterial biofilms due to the design and alteration in surface characteristics of dental implants [5]. Efforts using chemotherapeutic agents on implant surfaces have demonstrated positive outcomes in reducing bacterial counts, which appear to depend on the modification of the titanium surface and the selection of chemotherapeutic agents [6,7,8]. Further efforts are being attempted to address bacterial colonization by employing various biomaterials as carriers for antimicrobial agents or as coating materials on titanium implants. However, these attempts have yielded diverse outcomes, primarily due to concerns regarding potential toxicity, the need for optimizing coatings, and making physiochemical modifications [9].

Enamel matrix proteins, commercially available as Emdogain (Institute Straumann AG, Basel, Switzerland) are an FDA approved, widely recognized, and well-documented biomaterial that has been clinically and histologically proven to enhance periodontal regeneration [10,11,12]. Several cellular and molecular mechanisms have been attributed to enamel matrix proteins (EMP) in vitro. These mechanisms provide partial explanations for the biological effects observed in periodontal regeneration [13]. Clinical application of Emdogain^®^ has resulted in enhanced healing of soft tissues and reduced inflammation of the operated areas during post-operative period. One potential factor that could contribute to this early wound healing is its antimicrobial effect [14]. Despite being acknowledged for their regenerative potential, there are conflicting reports regarding the antimicrobial efficacy of EMPs [15,16].

Over the last decade, there has been extensive use of the second-generation platelet concentrate platelet-rich fibrin (PRF) owing to its significant advantage over plasma-rich protein, which includes development of stronger and more stable fibrin matrix which supports tissue healing and regeneration [17]. Additionally, the presence of leukocytes, platelets, and stem cells in PRF, which are host immune cells, are capable of impeding infectious pathogens [18,19]. PRF’s ability to form a beneficial fibrin clot is achieved through high relative centrifugation force (RCF) during preparation [20]. Sequentially, diverse protocols for PRF preparations have been established through adjustments in the centrifugation speed. This led to the development of advanced PRF (A-PRF+), achieved by employing lower RCF, thereby augmenting the platelet and leukocyte content. These components exhibit the capacity to release growth factors, which in turn contribute to their antimicrobial effect [21]. Further reduction in RCF resulted in development of i-PRF, a liquid which can be used either alone or in combination with other biomaterials encouraging cellular functions and growth factor release for tissue repair [22,23].

Recent research on the application of a PRF variant on SLA titanium surfaces has shown a significant reduction in bacterial counts, possibly due to the antimicrobial action of platelets [24]. Furthermore, a systematic review based on the antimicrobial effects of PRF reported that these autologous variants possess inhibitory effect against pathogens due to the release of platelets and its preparatory protocols [25].

Taking into account the available literature, there still seems to be a void in our understanding in the clinical preferences of these popular biologics. Hence, the objective of the present in vitro study was to investigate and compare the antimicrobial efficacy of two PRF variants, i.e., i-PRF and A-PRF+, with commercially available Emdogain™ coated on decontaminated smooth and rough Ti discs. It addresses the hypothesis that conditioning the Ti discs (smooth and rough) with any of these experimental biologics may not influence significant difference in the antimicrobial effectiveness, which primarily contributes to and supports peri-implant therapy.

## 2. Materials and Methods

**Figure 1:**
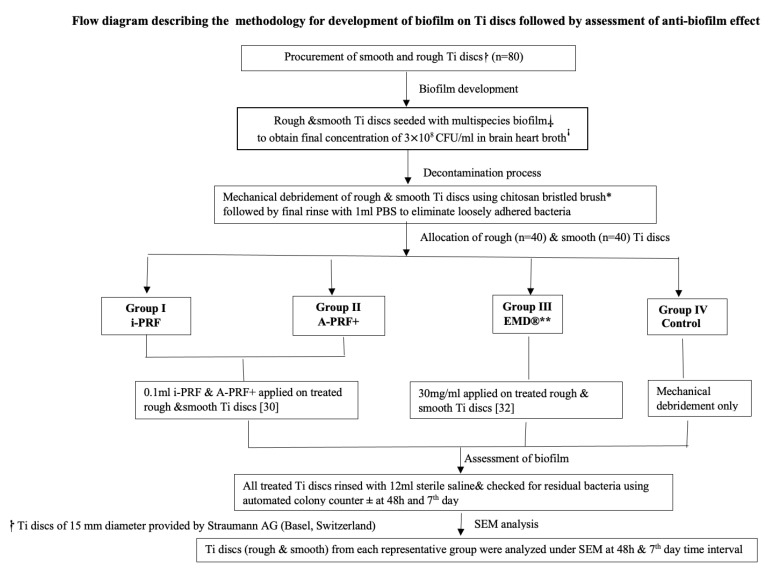
Flow diagram describing the methodology for development of biofilm on Ti discs followed by assessment of anti-biofilm effect. **⸷** Ti discs of 15 mm diameter provided by Straumann AG (Basel, Switzerland). ⸸ multispecies biofilm consisting of *Porphyromonas gingivalis* ATCC 33277™, *Prevotella intermedia* ATCC 15033™, *Tannerella forsythia* ATCC 4304™, *Treponema denticola* ATCC 35405™, *Actinomyces oris* 27044™, *Streptococcus anginosus* 3397™, and *Streptococcus oralis* 10557™. **ꜞ** Sigma-Aldrich, Merck KGaA, Darmstadt, Germany. * Chitosan-bristled brush inserted into handpiece (average speed 700 rpm) by Labrida Bioclean, Institut Straumann AG, Peter Merian Basel, Switzerland. ** Straumann AG Basel, Switzerland. ± BioMérieux Inc, Hazelwood, MO, USA.

The methodology detailing the complete process of evaluating antimicrobial efficacy is outlined in Figure 1.

### 2.1. Development of Biofilm on Ti Discs

**Figure 2:**
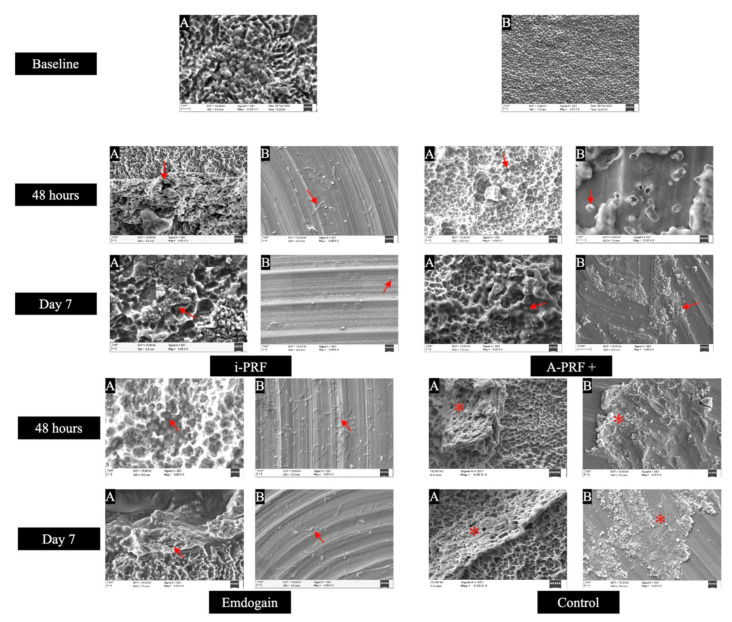
SEM (JSM6010, JEOL, Tokyo, Japan) representative images of all the experimental groups at 48 h and 7th day time interval. Magnifications of 5× and 10 KV were utilized. (A,B) Rough- and smooth-treated Ti discs in each experimental group. The i-PRF group revealed numerous bacterial cells trapped (→) within the matrix of rough Ti discs; however, smooth discs had reduced microbial colonies. A-PRF+ group displayed bacterial cells entangled (→) in the matrix of both rough and smooth Ti discs. The Emdogain group showed a higher presence of bacterial cells entrapped (→) in the lacunae of EMD-coated discs, particularly on rough discs, compared to smooth discs where fewer bacterial cells were observed. Control group revealed residual bacterial colonies (*) accumulated on both rough and smooth Ti discs.

Commercially available pure grade 4 titanium (Ti6Al4V) smooth and rough (sand-blasted followed by acid-etching) discs of 15 mm diameter were provided by Straumann AG (Institute Straumann AG, Basel, Switzerland). All the discs were inoculated with a multispecies biofilm consisting of *Porphyromonas gingivalis* ATCC 33277™, *Prevotella intermedia* ATCC 15033™, *Tannerella forsythia* ATCC 4304™, *Treponema denticola* ATCC 35405™, *Actinomyces oris* 27044™, *Streptococcus anginosus* 3397™, and *Streptococcus oralis* 10557™. The methodology followed for biofilm development on the Ti discs was adapted from research conducted by Shen et al. [26]. In brief, bovine dermal type I collagen (10 μg/mL collagen in 0.012 M HCl in water (Gibco, Fisher Scientific, Massachusetts, MA, USA) was applied on the Ti discs and stored overnight at 4 °C. This step was undertaken to mimic the elimination of inherent bone components occurring in peri-implantitis due to the process of osteoclastic resorption. The pre-coated Ti discs were positioned in 24-well tissue culture plates with 2 mL of bacterial suspension. The plates were then placed in an anaerobic environment at 37 °C for 21 days, with weekly renewal of the suspension. All bacterial strains were cultured to achieve a final concentration of approximately 3.0 × 10^8^ CFU/mL in brain–heart broth (Sigma-Aldrich, Merck KGaA, Darmstadt, Germany). All the discs were checked for adherent bacteria colony-forming units (CFU) with the aid of the automated colony counter (Analytik Jena, CA, USA). To confirm the development of the mature polymicrobial biofilm, one Ti disc of each type (both smooth and rough) was subjected to scanning electron microscopic (SEM) analysis (JSM6010, JEOL, Tokyo, Japan) and representative images were taken (Figure 2). A standard working distance of 5–10 mm using magnifications from 5000× to 20,000× were utilized.

### 2.2. Automated Colony Counting for Microbial Colonies

Automated counting of the bacterial colonies was performed using UVP Colony Doc-It imaging station (Analytik Jena US, Upland, CA, USA) with the vision works capture and analysis software 9.1 (Analytik Jena US). The pictures of the tissue culture plates with bacterial suspension were captured with the high-quality standard setting, the doors of the imaging station closed with an overhead white light, and a black background. The lids of the culture plates were removed with the culture medium surface facing upwards towards the camera. Subsequently, pictures of the plates with the results of the automated colony counting were visually observed on a computer display.

### 2.3. Decontamination Process

Effective debridement is crucial for achieving and maintaining long-term stability in periodontal health. Hence, subsequent to the development of multispecies biofilm on the Ti discs, mechanical debridement was performed on all the discs (rough and smooth) using a chitosan-bristled brush inserted into an oscillating handpiece (average speed—700 rpm, Labrida Bioclean, Institut Straumann AG, Peter Merian, Basel, Switzerland). Gamma sterilization was also performed after cleaning with the chitosan-bristled brush. The debridement efficacy was assessed through morphologic analysis of both contaminated and decontaminated disc surfaces using SEM (JSM6010, JEOL, Tokyo, Japan).

### 2.4. Preparation of Biologic Constituents Required for the Study

This section involved preparation of PRF variants, i.e., A-PRF+ and i-PRF. The blood samples were collected from six healthy volunteers, aged 25 to 35 years. The study was approved by the Institutional Review board (IEC1:24/2022) which was in accordance with the Declaration of Helsinki. The volunteers were provided with comprehensive information regarding the study and gave informed consent prior to the blood donation process. The PRF preparation was as per the protocol followed by Kobayashi et al. [27]. For A-PRF+, two tubes with 10 mL of whole blood were centrifuged (Duo^®^Quattro centrifuge, Avtec surgical, Mount Pleasant, SC, USA) at 1300 rpm/208 g × 8 min. The centrifugation process followed the manufacturer’s instructions. After the designated centrifugation steps, the tubes were kept vertical for 5 min [28]. The fibrin clot located in the middle part of the tube, between the red corpuscular phase at the bottom of the tube and the acellular plasma at the top, was used for the study [29]. To prepare i-PRF, two tubes with 10 mL of whole blood were transferred into a vacutainer without anticoagulant, and the mixture was promptly centrifuged at 700 rpm for 3 min. Subsequently, 1 mL was extracted from the upper layer of the resulting fibrin clot, serving as the designated i-PRF. Then, the formed i-PRF was applied into plastic culture dishes with 5 mL of Dulbecco’s Modified Eagle Medium (Sigma-Aldrich Chemie GmbH, Taufkirchen, Germany) and processed [23].

### 2.5. Application of Experimental Agents

All the decontaminated Ti discs were rinsed with 1 mL sterile phosphate buffer saline (PBS). Following this, the experimental agents were applied on the discs as per the assigned groups. The study comprised four experimental groups, each consisting of 10 Ti roughened surface discs and 10 Ti smooth surface discs, totaling 80 discs. The groups were designated as follows: (a) Group I-i-PRF-, (b) Group II-A-PRF+, (c) Group III-EMD (Emdogain^®^), and (d) Group IV—control (only mechanical debridement) discs. The methodology for evaluation of anti-biofilm effect was adapted from the work performed by Schuldt et al. [24]. For the application of the biologic agents, 0.1 mL of i-PRF and 0.1 mL of A-PRF+ were coated separately on the surface of the Ti discs (both smooth and rough from both groups I and II). The concentration was chosen considering its antibacterial effect, as per the study conducted by Karde et al. [30]. Further, these Ti discs were inserted into the culture plate wells containing 2 mL antibiotic and serum free DMEM (Dulbecco’s Modified Eagle Medium) under aerobic conditions at 37 °C for 48 h. In Group III, 30 mg/mL of EMD (Straumann AG, Basel, Switzerland) was used based on the relevant literature documented in previous studies [31,32]. And Group IV served as control, wherein the discs were debrided mechanically without the use of biological agents. After incubation, the smooth and rough discs in all the experimental groups were rinsed with 12 mL of sterile saline. The complete antimicrobial experimental process was evaluated at two time points, i.e., after 48 h and after 7 days. Further, post application of experimental agents, the Ti discs from each group (both smooth and rough) were analyzed using SEM at the scheduled study time period and representative images were taken (Figure 2).

### 2.6. Statistical Analysis

The data compiled from the antimicrobial estimation were statistical analyzed using IBM SPSS software (IBM SPSS Statistics for Windows, version 23.0; IBM Corp., Armonk, New York, NY, USA). Intergroup comparison of CFU values at each time point was measured using Kruskal–Wallis test. The Mann–Whitney ‘U’ test was utilized for pairwise intergroup comparison of CFU values at different time points. In each study group, comparison of CFU counts at study time intervals was carried out using the Friedman test. Subsequently, the Wilcoxon signed-rank test was used for pairwise comparison. The probability level was set at 5% to assess the statistical significance of the differences between the experimental groups.

## 3. Results

At baseline, the microbial count on all Ti discs were standardized to 3 × 10^8^ CFU/mL (8.48 log_10_ CFU). A significant decrease in microbial scores were observed across all experimental groups when compared to their initial baseline values, which is attributed to the mechanical debridement conducted on all discs. Among the study groups, the control group (mechanical debridement only) exhibited the highest number of microbial colonies both at 48 h and after the 7 day study period. Between the experimental agents, Group I (i-PRF) demonstrated the maximum CFU reduction compared to other experimental groups (*p* < 0.001) (Table 1). The mean intergroup comparison of CFU values at 48 h and 7 days revealed a statistically significant difference for both rough and smooth Ti discs (*p* < 0.001). In all the experimental groups, smooth Ti discs displayed a greater reduction in microbes compared to rough Ti discs when treated (Table 1).

**Figure 3:**
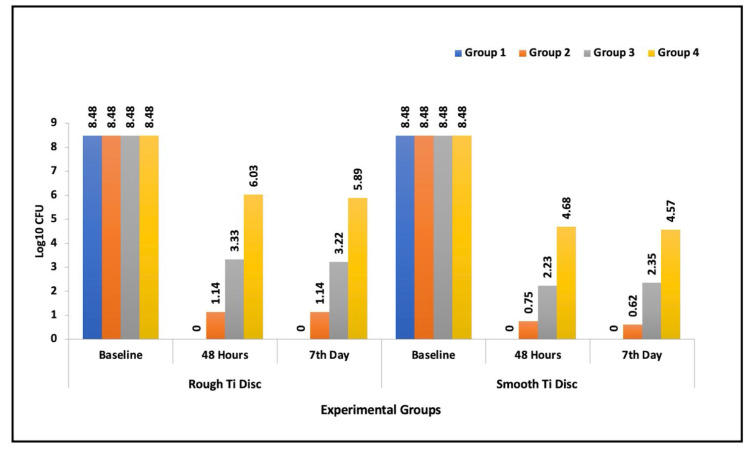
Bar graph exhibiting the antimicrobial efficacy of the experimental groups on Ti discs (rough and smooth) at the specified study time periods.

Table 2 summarizes pairwise comparison between the study groups (inclusive of both smooth and rough Ti discs), the results revealed statistically significant differences both at 48 h and 7 days. Group I (i-PRF) performed the best in terms of anti-biofilm (*p* = 0.03 and *p* = 0.001). Similarly, Group II (A-PRF+) also exhibited a statistically significant CFU reduction relative to Group III (EMD). Considering the antimicrobial efficacy of each experimental group within the study time points, the major reduction in CFU values was noted from baseline to the 7th day (Figure 3). However, the time span from 48 h to 7 days did not display a significant difference in the microbial reduction (Table 3). In conclusion, the results of the study revealed that i-PRF performed superior antimicrobial activity compared to A-PRF, followed by EMD.

### Description of Scanning Electron Microscopy (SEM) Images

At baseline, significant accumulation of microbial species was observed for both rough and smooth Ti discs. At 48 h and 7 days, groups I (i-PRF), II (A-PRF+), and III (EMD) demonstrated bacterial cells entrapped within the matrices of the experimental agents (i-PRF, A-PRF+, and EMD), primarily on the roughened discs. Finally, Group IV revealed remnants of bacterial accumulation on both discs (smooth and rough) of the control groups at the mentioned study time points. Representative SEM images are displayed in Figure 2 (A,B).

## 4. Discussion

The purpose of this study was to assess and compare the impact of two autologous platelet-rich fibrin formulations (i-PRF and A-PRF+) as well as EMD on titanium discs previously contaminated with a multispecies biofilm. We observed an anti-biofilm effect in all experimental groups, with the maximum reduction in bacterial counts occurring at 48 h and persisting throughout the study. Notably, the i-PRF medium applied to smooth- and rough-treated discs showed the most significant reduction in microbial colonies. The substantial effectiveness of i-PRF could be attributed to the presence of antimicrobial peptides, which could enhance its ability to combat biofilms [33]. i-PRF has shown superior release of growth factors (PDGF-AA, PDGF-AB, EGF, and IGF-1) over a 10-day period compared to first-generation platelet concentrates [23]. The inclusion of leukocytes, platelets, and stem cells in PRF, which are integral components of the host’s immune system, can aid in inhibiting infectious pathogens. Furthermore, PRF provides a stronger and more stable fibrin matrix that supports tissue healing and regeneration [17,18,19]. Previous research has shown that antimicrobial activity of i-PRF peaks at 48 h post-application [30]. This finding was consistent with our study, which also observed the superior antimicrobial effect at 48 h, sustained at the same level for 7 days. Another possible explanation for these favorable results could be due to the low-speed centrifugation protocol for preparation of i-PRF which resulted in release of ample growth factors [23,34]. i-PRF also proved effective against key pathogens compared to traditional variants like PRF and PRP, suggesting its practicality for periodontal regeneration and wound healing [35].

A-PRF+ preparations exhibit a steady release of PDGF-AA over a span of 10 days, distinguishing them from other PRF variants [36]. While several studies have demonstrated the advantageous antimicrobial impact of A-PRF+ on periodontopathogens, it is generally not as remarkable as that observed with i-PRF [37,38]. Similar results were reported in the present study, where A-PRF+ significantly retarded microbial growth, however at a lower effect than that of i-PRF.

Another extensively used preparation in periodontal defects is Emdogain (EMD), which contains enamel matrix proteins as a stimulus for recruiting numerous native progenitor cells. Initial studies assessing the effects of EMD on biofilm models revealed a significant effect on plaque vitality [16]. However, this activity was attributed to the propylene glycol alginate (PGA) vehicle and its acidic pH rather than any inherent antimicrobial factors present in EMD. Consequently, biofilm models have shown EMD to possess an inferior antimicrobial impact, as seen previously [15,39]. These data aligns with our findings where EMD exhibited a modest antimicrobial effect which was lower compared to both tested autologous platelet concentrates. Even though the literature has quoted high survival rates, dental implants are still receptive to complications like peri-implantitis. One of the leading reasons for the low success rates is the rough implant surface that encourages the growth of biofilm. Hence, meticulous decontamination of rough Ti surfaces is integral for the efficacious treatment of peri-implant diseases. Studies using chemotherapeutic agents for biofilm elimination from implant surfaces have failed to display complete removal of toxins [40]. Therefore, based on the findings of this study, a significant advantage of implants coated with autologous platelet concentrates (i-PRF, A-PRF+) is their effective decontamination of the implant surface. Additionally, they are readily available and cost-effective. They can serve as a substitute for enamel matrix derivative and other commonly used chemical plaque control agents. An inherent limitation of the present study is its in vitro design. The prepared biofilm may be different in composition to actual subgingival films formed at peri-implantitis sites. Another relevant factor could be the composition and efficacy of platelet concentrates, which vary greatly based on gender, age, systemic status, smoking status, ethnicity, and diet [41]. Based on the results of this research work, potential future advancements in periodontal therapy may be strategized. The combination of i-PRF and Emdogain for periodontal regeneration and tissue healing shows promise. These biologics could complement each other owing to its beneficial properties, potentially leading to enhanced periodontal tissue regeneration, improved antimicrobial effects, and increased stability of the fibrin matrix. Future research may focus on developing standardized protocols for combining i-PRF with Emdogain in clinical practice, including application techniques, determining the ideal ratio, and optimal timing for treatment.

## 5. Conclusions

Based on the current findings, we conclude that i-PRF possessed superior antimicrobial efficacy on the coated Ti disc surfaces compared to A-PRF+ and EMD. This benefit, combined with the fundamental advantages of autologous platelet concentrates, promote i-PRF as a suitable agent for chair-side implant surface decontamination. These findings also provide valuable insights to clinicians on the preference of implant coating that can reduce risk of peri-implant infections and thereby improve patient care. However, this research work also visualized the antimicrobial capabilities of EMD, suggesting that use of both i-PRF and EMD as valuable bioactive materials can be recommended to improve tissue healing effect. However, all these outcomes need to be verified by further research, and clinical trials are necessary to fully explore the benefits and establish best practices.

## Figures and Tables

**Table 1:** Comparison of log_10_ CFU between the study groups at each time interval using Kruskal–Wallis test. * *p* < 0.05 significant.

		Group	N	Mean	SD	Min	Max	Percentiles			Kruskal–Wallis Test	
								Q1	Median	Q3	Chi Square Value	*p*-Value
Rough Ti discs	Baseline	I	10	8.48	0	8.48	8.48	8.48	8.48	8.48	0	1.00 (NS)
		II	10	8.48	0	8.48	8.48	8.48	8.48	8.48		
		III	10	8.48	0	8.48	8.48	8.48	8.48	8.48		
		IV	10	8.48	0	8.48	8.48	8.48	8.48	8.48		
	48 h	I	10	0	0	0	0	0	0	0	37.26	<0.001 *
		II	10	1.14	0.26	0.85	1.60	0.98	1.00	1.35		
		III	10	3.33	0.46	2.30	3.90	3.00	3.48	3.65		
		IV	10	6.03	0.69	5.00	6.78	5.30	6.30	6.60		
	7 day	I	10	0	0	0	0	0	0	0	37.34	<0.001 *
		II	10	1.14	0.19	1.00	1.48	1.00	1.00	1.30		
		III	10	3.22	0.68	2.00	3.78	2.75	3.48	3.65		
		IV	10	5.89	0.47	5.48	6.78	5.48	5.74	6.15		
Smooth Ti discs	Baseline	I	10	8.48	0	8.48	8.48	8.48	8.48	8.48	0	1.00 (NS)
		II	10	8.48	0	8.48	8.48	8.48	8.48	8.48		
		III	10	8.48	0	8.48	8.48	8.48	8.48	8.48		
		IV	10	8.48	0	8.48	8.48	8.48	8.48	8.48		
	48 h	I	10	0	0	0	0	0	0	0	30.04	<0.001 *
		II	10	0.75	0.69	0	1.78	0	0.95	1.35		
		III	10	2.23	0.44	1.48	3.00	2.00	2.15	2.55		
		IV	10	4.68	1.90	0	6.78	4.18	5.15	5.78		
	7 day	I	10	0	0	0	0	0	0	0	30.39	<0.001 *
		II	10	0.62	0.54	0	1.30	0	0.95	1.00		
		III	10	2.35	0.34	2	2.95	2.00	2.39	2.60		
		IV	10	4.57	1.73	0	5.60	4.45	5.00	5.60		

**Table 2:** Pairwise comparison of log_10_ CFU between the study groups at each time interval using Mann–Whitney test. * *p* < 0.05 significant. NS: not significant.

	Groups	Rough Ti Discs		Smooth Ti Discs	
		U Statistic	*p*-Value	U Statistic	*p*-Value
Baseline	I vs. II	50	1.00 (NS)	50	1.00 (NS)
	I vs. III	50	1.00 (NS)	50	1.00 (NS)
	I vs. IV	50	1.00 (NS)	50	1.00 (NS)
	II vs. III	50	1.00 (NS)	50	1.00 (NS)
	II vs. IV	50	1.00 (NS)	50	1.00 (NS)
	III vs. IV	50	1.00 (NS)	50	1.00 (NS)
48 h	I vs. II	0	<0.001 *	20	0.03 *
	I vs. III	0	<0.001 *	0	<0.001 *
	I vs. IV	0	<0.001 *	5	<0.001 *
	II vs. III	0	<0.001 *	1.5	<0.001 *
	II vs. IV	0	<0.001 *	8	0.006 *
	III vs. IV	0	<0.001 *	10	0.012 *
7 day	I vs. II	0	<0.001 *	20	0.03 *
	I vs. III	0	<0.001 *	0	<0.001 *
	I vs. IV	0	<0.001 *	5	<0.001 *
	II vs. III	0	<0.001 *	0	<0.001 *
	II vs. IV	0	<0.001 *	8	0.006 *
	III vs. IV	0	<0.001 *	10	0.012 *

**Table 3:** Comparison of log_10_ CFU between different time intervals in each study group using Wilcoxon signed-rank test, * *p* < 0.05 significant. NS: not significant.

	Group		48 h—Baseline	Day 7—Baseline	Day 7—48 h
Rough Ti discs	I	Z	−3.16	−3.16	0
		*p*-value	0.006 *	0.006 *	1.00 (NS)
	II	Z	−2.82	−2.88	−0.14
		*p*-value	0.02 *	0.01 *	0.89 (NS)
	III	Z	−2.81	−2.82 *	−0.24
		*p*-value	0.02 *	0.02 *	0.81 (NS)
	IV	Z	−2.82	−2.81	−0.67
		*p*-value	0.02 *	0.02 *	0.51 (NS)
Smooth Ti discs	I	Z	−3.16	−3.16	0
		*p*-value	0.006 *	0.006 *	1.00 (NS)
	II	Z	−2.82	−2.84	−1.75
		*p*-value	0.02 *	0.02 *	0.08 (NS)
	III	Z	−2.82	−2.83	−0.85
		*p*-value	0.02 *	0.02 *	0.40 (NS)
	IV	Z	−2.81	−2.83	−0.30
		*p*-value	0.02 *	0.02 *	0.77 (NS)

## Data Availability

The complete dataset has been presented in this study.
